# Simpler, Faster, and Sensitive Zika Virus Assay Using Smartphone Detection of Loop-mediated Isothermal Amplification on Paper Microfluidic Chips

**DOI:** 10.1038/s41598-018-30797-9

**Published:** 2018-08-20

**Authors:** Kattika Kaarj, Patarajarin Akarapipad, Jeong-Yeol Yoon

**Affiliations:** 1 Department of Biosystems Engineering, The University of Arizona, Tucson, Arizona 85721 USA; 2 Department of Biomedical Engineering, The University of Arizona, Tucson, Arizona 85721 USA

## Abstract

The recent Zika virus (ZIKV) outbreak has prompted the need for field-ready diagnostics that are rapid, easy-to-use, handheld, and disposable while providing extreme sensitivity and specificity. To meet this demand, we developed a wax-printed paper microfluidic chip utilizing reverse transcription loop-mediated isothermal amplification (RT-LAMP). The developed simple and sensitive ZIKV assay was demonstrated using undiluted tap water, human urine, and diluted (10%) human blood plasma. Paper type, pore size, and channel dimension of various paper microfluidic chips were investigated and optimized to ensure proper filtration of direct-use biological samples (tap water, urine, and plasma) during capillary action-driven flow. Once ZIKV RNA has flowed and reached to a detection area of the paper microfluidic chip, it was excised for the addition of an RT-LAMP mixture with a pH indicator, then placed on a hot plate at 68 °C. Visible color changes from successful amplification were observed in 15 minutes and quantified by smartphone imaging. The limit of detection was as low as 1 copy/μL. The developed platform can also be used for identifying other flaviviruses, such as Chikungunya virus (CHIKV) and Dengue virus (DENV), and potentially other quickly transmitted virus pathogens, towards field-based diagnostics.

## Introduction

Zika virus (ZIKV) outbreak has become a major global health concern since 2015–2016, due to its rapid, worldwide spread and serious concerns of microcephaly in newborn babies^1–4^. ZIKV is transmitted by infected mosquitos (*Aedes aegypti* and *Aedes albopictus*), and can cause symptoms such as fever, headache, rash, and joint/muscle pain, which can be relatively mild (except in newborn babies)^2^. In fact, ZIKV shares the same symptoms and the same genus of mosquito vector with other flaviviruses including Chikungunya virus (CHIKV) and Dengue virus (DENV)^3^ making them difficult to identify based on symptoms. A rapid, specific, and sensitive method of detecting ZIKV (and other flaviviruses) is essential for providing adequate and accurate treatment, managing and tracking ZIKV in pregnant women and their fetuses, ensuring the safety of blood transfusion, and eventually monitoring and controlling the spread of the virus.

A couple of diagnostic methods have already been commercialized for ZIKV (and other flaviviruses). The most popular platforms are antibody-based assays, typically utilizing IgM antibodies, including enzyme-linked immunosorbent assay (ELISA) kits and lateral flow assays (LFAs; also known as rapid tests)^5,6^. While they are relatively simple and rapid, they can only detect high antigen concentrations, corresponding to the later stage of infection^7^. Moreover, this IgM-based assay lacks sensitivity, especially in identifying species identification within flavivirus genus, e.g., ZIKV vs. CHIKV or DENV^5,6^. A better alternative, recommended by the U.S. Centers for Disease Control and Prevention (CDC), is reverse transcription quantitative real-time polymerase chain reaction (RT-qPCR)^5^. Although this method is much more specific and sensitive than antibody-based assays, the patient’s sample (i.e., urine or blood) must be pre-treated and purified, as many components in urine and blood are PCR inhibitors^8^. Taken together, RT-qPCR is inappropriate for field- and clinical-ready detection of ZIKV and other flaviviruses, due to its requirement of laboratory environment, equipment, and trained professionals.

Recently, a simpler version of nucleic acid amplification has emerged requiring only one temperature, rather than three temperatures needed for conventional PCRs. One such example is loop-mediated isothermal amplification (LAMP). In this case, we are using RT-LAMP as we are interested in detecting RNA viruses. LAMP uses 4 to 6 primers that bind to the particular regions on the nucleic acid strands. Rather than splitting the extended products at high denaturation temperature in preparation for the next cycle, loop structures are formed at the end of the amplified products, allowing the next stage of primer annealing and subsequent extension, all occurring at the same temperature. It is highly specific (4–6 vs. 2 primers) and requires only a single temperature (typically 60–70 °C), enabling the process to be simple and rapid^9^. RT-LAMP is a promising and robust approach for ZIKV and other flavivirus diagnostics, as it is simple and rapid, thus appropriate for field diagnostics while providing extremely high sensitivity and specificity^5,9^.

Several papers have recently been published demonstrating RT-LAMP for virus detection, including ZIKV. Song *et al*.^10^ developed a chemically heated cup to provide the constant temperature needed for RT-LAMP towards a field-ready device, although their results showed some nonspecific amplification due to uneven heating. Lee *et al*.^11^ demonstrated the use of both RT-LAMP and LFA. While the amplicons were identified on the LFA strips in a handheld manner, RT-LAMP reactions were still conducted using a conventional, laboratory-based method. Furthermore, the demonstrated colorimetric detection was based on a streptavidin-biotin binding mechanism on gold nanoparticles, potentially causing non-specific cross-binding with other molecules and/or targets.

While many different colorimetric and fluorescent dyes are available for quantifying LAMP, such as leuco crystal violet (LCV) and fluorescent intercalating dyes, they typically require multiple steps, additional chemical substances, a closed container or dark environment, and a specific excitation light source^10,12,13^. For field applications, a simpler type of dye is needed that is affected by neither ambient lighting perturbations nor the presence of other amplification-inhibiting molecules in the sample. For this purpose, pH indicator-based colorimetric assay kits could be used (e.g., WarmStart colorimetric LAMP master mix by New England Biolabs), which includes a pH indicator (phenol red) in the master mix reagent, requiring no additional step, no excitation light source, and no closed container (identifiable under ambient light)^14^.

In fact, such pH indicator-based assays can further be improved in sensitivity and reproducibility with digital cameras (complementary metal oxide semiconductor or CMOS array), including smartphone cameras. While smartphone camera detection has been demonstrated for RT-LAMP, only fluorescence detection has been demonstrated^12,13^, which requires an additional light source, optical filters, and a closed container. In addition, RT-LAMP reactions were still conducted in a conventional, laboratory-based manner in these studies, making them difficult to be used for field applications.

To facilitate and improve field-ready ZIKV diagnostics, we aim to demonstrate RT-LAMP on a simple and disposable platform, coupled with a pH indicator-based colorimetric assay, a simple hot plate, and subsequent smartphone detection, thus forgoing an excitation light source, optical filters, and a closed container. As a simple and disposable platform, we chose a paper microfluidic chip, since it is flexible, inexpensive, easy-to-fabricate by simple wax printing, and thus optimum for field use. Paper also provides spontaneous flow via capillary action (also known as wicking) without the need for an external pump or pumping mechanism. In addition, we can also expect filtration (or at least longer retention time) of large-sized molecules in urine or blood plasma samples within paper fibers, potentially allowing samples to be used with minimum pre-treatment. Cellulose fibers in paper, due to its strong negative polarity, are well-suited to separate target RNAs and their amplified products (strong negative charges) from other proteins and cell fragments (much weaker negative charges). As a result, the target RNAs are pushed toward the end of the channel along with the bulk liquid flow, while the proteins and cell fragments are slowed down with increased retention time^15–17^. Finally, a smartphone is used to quantify the normalized intensities from the pH indicator dye to encompass a handheld and reproducible assay^18^. The paper microfluidic platform also enables to conduct sample filtration, subsequent amplification, and real-time quantification, all together on a single paper microfluidic chip, without any liquid transfers. Therefore, the possibility of introducing contaminants from the environment can significantly be reduced, which is a common problem in conventional LAMP assays.

**Figure 1:**
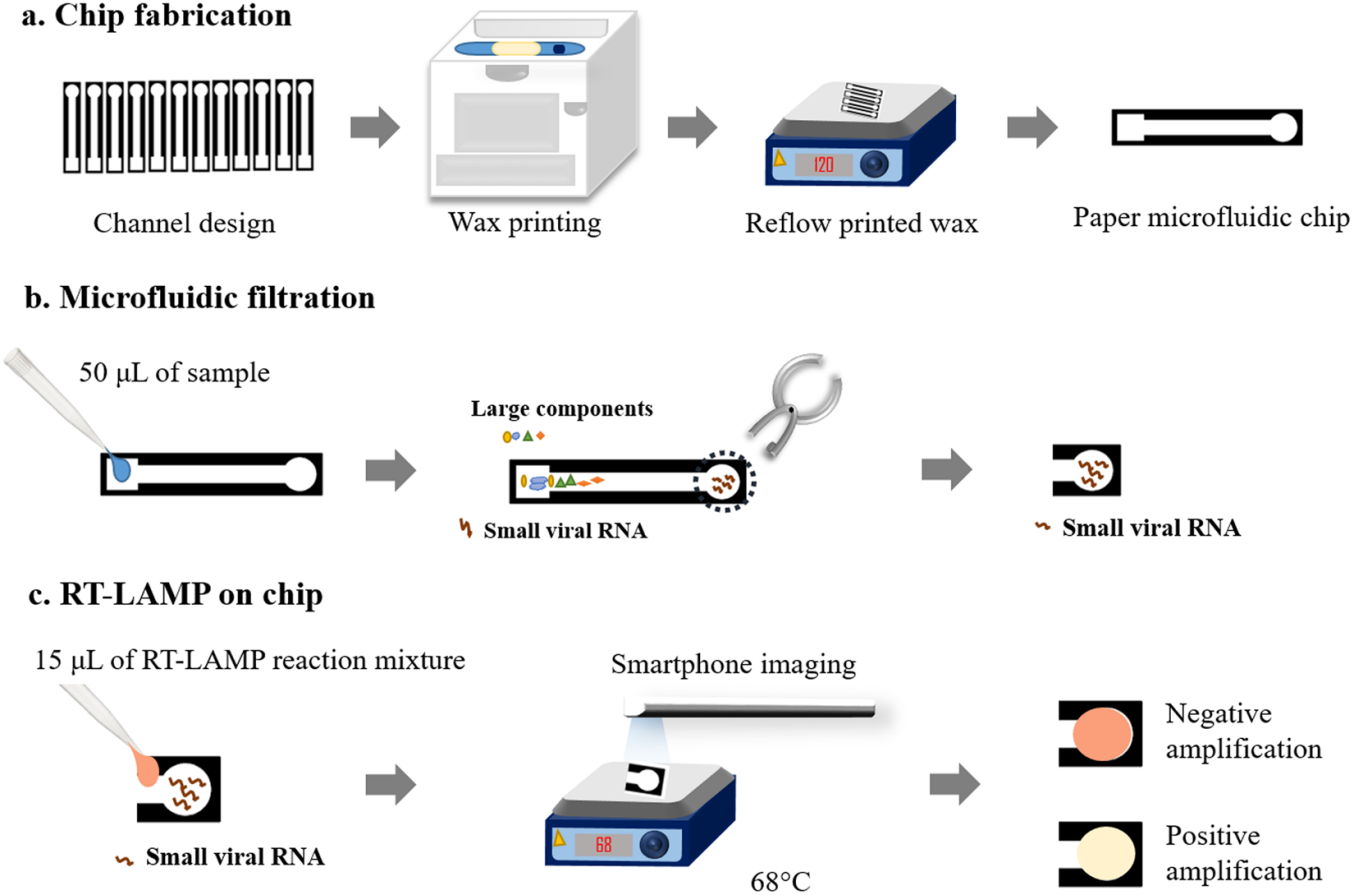
Schematic of paper microfluidic RT-LAMP assay. **(a)** The paper microfluidic chips were designed with SolidWorks and wax-printed on cellulose grade 4 paper. Printed wax patterns were melted and vertically flown through the depth of paper by placing the chips on a hot plate. The dark wax area represents the hydrophobic barrier and the bright area the hydrophilic area where liquid flows. **(b)** ZIKV-spiked samples of deionized water, tap water (undiluted), human urine (undiluted), or human blood plasma (diluted to 10% with PlasmaLyte), were loaded onto the loading area of a paper microfluidic chip and spontaneously flowed through the channel via capillary action. Contaminants in the sample matrices were filtered during this process, while target RNA flowed at the same speed of bulk liquid. **(c)** The circular end (detection area) of the paper was cut, and the RT-LAMP reaction mixture was added onto it. This cut-out paper was placed on a hot plate at 68 °C up to 40 minutes to achieve amplification. The color change of the paper is monitored *in situ* through capturing images with a smartphone.

Toward this aim, we optimized the set temperature of a hot plate that delivers the optimum RT-LAMP temperature to paper microfluidic chips (Fig. 1), as well as the paper type and pore size. Coloration was monitored using a smartphone camera from 5 to 40 minutes for real-time quantification of RT-LAMP. Specificity tests were also conducted using influenza A/H1N1 that has a similar genome size to that of ZIKV. The limit of detection was also evaluated by serially diluting the sample. Finally, various sample matrices were tested, including tap water, human urine, and human blood plasma, towards field-ready diagnostics.

## Results and Discussion

### Optimization of temperature and paper type for RT-LAMP

**Figure 2:**
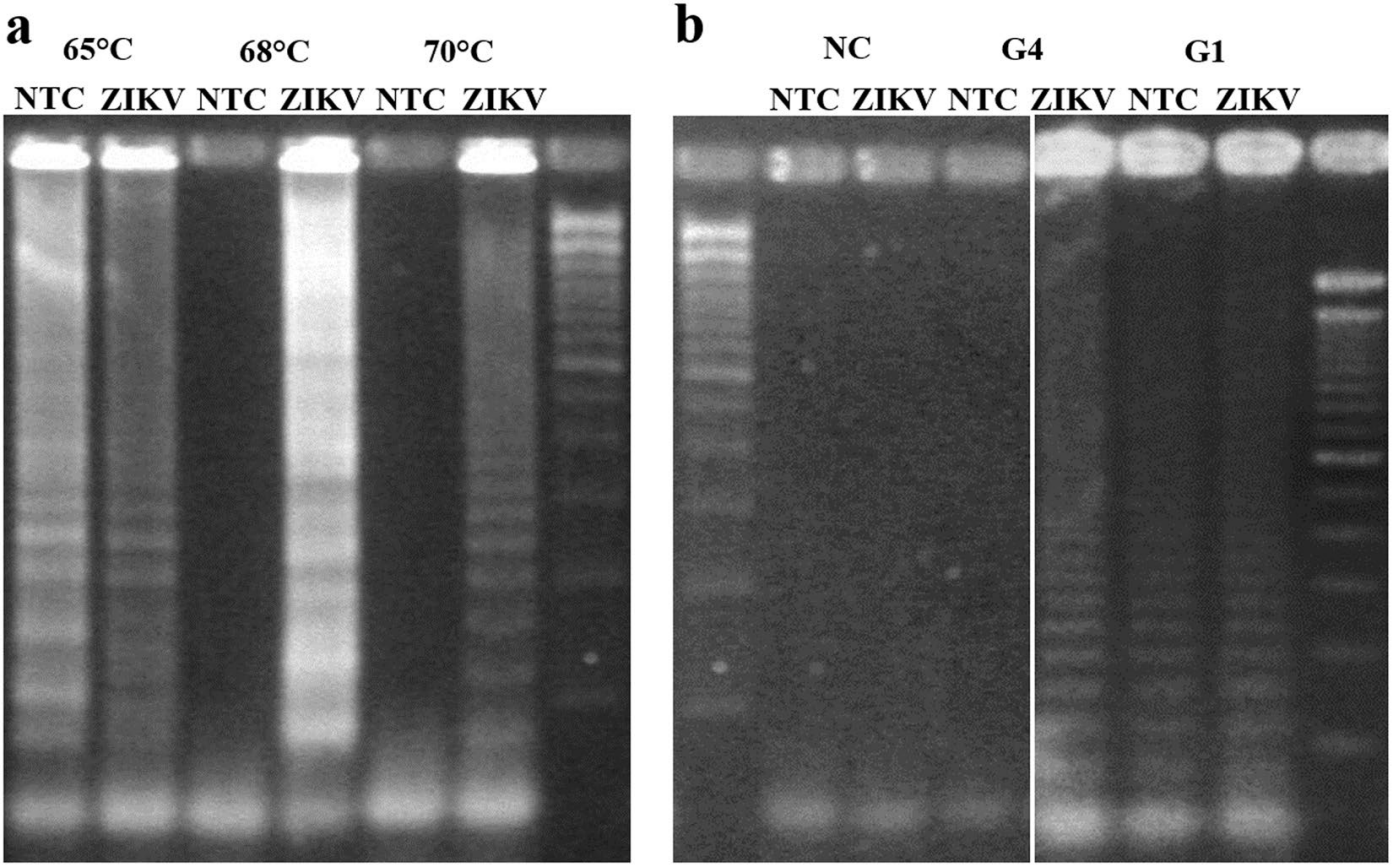
Optimization of temperature and paper type. **(a)** Temperature optimization among 65 °C, 68 °C and 70 °C using a conventional thermocycler, as confirmed with 3% w/v agarose gel electrophoresis. **(b)** Paper type optimization among NC (nitrocellulose), G4 (cellulose grade 4) and G1 (cellulose grade 1) papers, as confirmed with 3% w/v agarose gel electrophoresis.

Temperature is the most critical factor for a specific and efficient RT-LAMP reaction. Three varied temperatures, 65 °C, 68 °C and 70 °C, were investigated using a conventional thermocycler (for maintaining one temperature needed for RT-LAMP). Amplified products were confirmed by 3% w/v agarose gel electrophoresis (Fig. 2a). Both reverse transcription and LAMP reactions (i.e. entire reaction) were conducted at a single temperature in a single step. Non-specific amplification occurred at 65 °C in both control and target samples since the low temperature allows the primers to non-specifically bind to the target strand^19^. At 70 °C, no amplification reaction was found in both control and target samples, since the temperature is too high for primers to bind to the target template^19^. At 68 °C, the target template was successfully amplified from the target-contained sample, with no amplification from the no target control (NTC) sample. Therefore, 68 °C was selected as the optimum temperature for our RT-LAMP reaction, which was used throughout this study.

The choice of paper type is very important for conducting RT-LAMP on paper microfluidic chips. Three different types of paper were considered for amplifying ZIKV, including nitrocellulose (NC), grade 4 (G4) cellulose, and grade 1 (G1) cellulose paper, with varying pore size and molecular and fiber structure. ZIKV sample solutions were pipetted directly onto paper substrates, which had no microfluidic channels at this stage. The solution spread spontaneously over the paper fibers via capillary action. After successful spreading, the RT-LAMP reaction mixture consisting of primers and master mix was added. The paper was then sandwiched between two glass slides and sealed with Parafilm M to prevent evaporation. Amplification was initiated once this glass-sandwiched paper was placed onto a regular hot plate, pre-set to a temperature of 68 °C. After 30 minutes, the amplicons were eluted out of the paper by submersion in TE (Tris-HCl and EDTA) buffer, followed by 30 minutes of sonication. Amplification was then confirmed with gel electrophoresis of eluted amplicons. According to the results of the gel electrophoresis (Fig. 2), the G4 paper showed the best amplification result, followed by G1 paper showing weak amplification, then by NC showing no amplification. This trend corresponds very well to the pore sizes of the three different paper types: 20–25 μm of G4, 10 μm of G1, and 0.2–0.45 μm of NC paper. It should be noted that most NC papers come with smaller pore sizes than cellulose papers. While all three pore sizes are sufficiently bigger than the dimensions of whole ZIKV, amplified products, and other components of RT-LAMP reaction mixture, pore size does play an important role. The strong negative charge of NC paper, typically preferred in many different lateral flow assays (LFAs), did not generate successful amplification. Relatively weaker negative polarity of cellulose paper is deemed appropriate. In addition, as each pore in the detection area of a paper microfluidic chip functions as a single bioreactor, we can speculate such bioreactor should be as big as possible for successful RT-LAMP reaction, especially for the samples with low titers of nucleic acid templates. This small bioreactor concept may lead to the future possibility of conducting a digital droplet PCR in a much more affordable and simple set-up for handling and detection, if the pore size can further be decreased. However, such demonstration would require a different image analysis approach, for example, the use of a fluorescence microscopy attachment to a smartphone.

### Real-time monitoring and assay time

**Figure 3:**
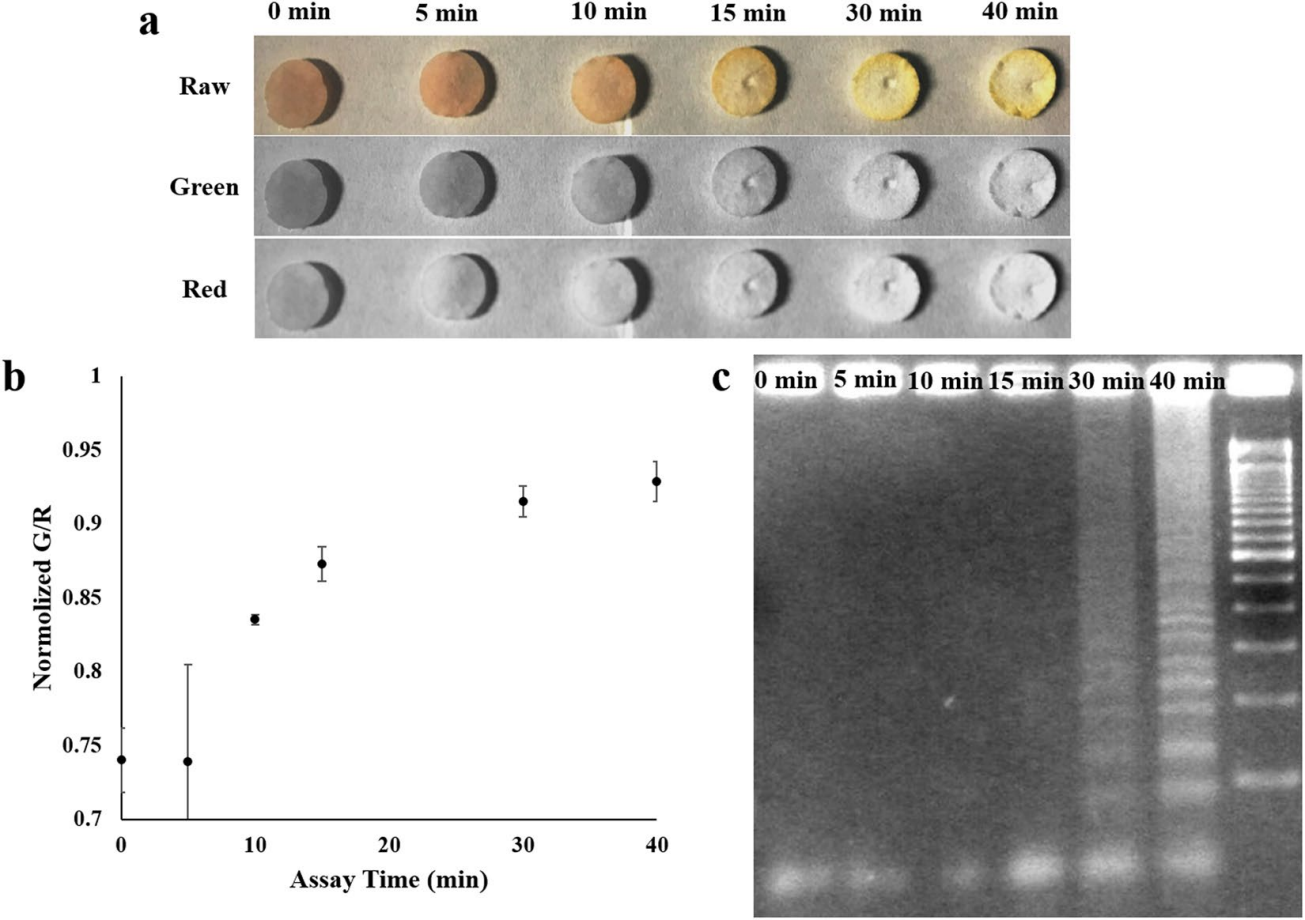
Real-time monitoring and assay time of paper microfluidic RT-LAMP assay. After paper microfluidic filtration of samples, the excised (hole-punched) detection area of the chip was added with RT-LAMP reaction mixture and placed on a hot plate to commence amplification. **(a)** Representative raw, green and red channel smartphone images captured at 0, 5, 10, 15, 30, and 40 minutes. **(b)** Normalized green to red (G/R) intensity values are plotted against time. Average of three different experiments, each using a different paper microfluidic chip, sample, and reagent. Error bars represent standard errors. **(c)** Amplifications were confirmed with 3% w/v agarose gel electrophoresis, using the eluted products from the paper chips, stained with ethidium bromide.

With the optimized temperature (68 °C) and paper type (G4), RT-LAMP reactions were repeated on a paper microfluidic chip (Fig. 1a) for detecting ZIKV (initial copy number = 10^3^ copies/µL), but now with the incorporation of a colorimetric dye to the reaction mixture. The ZIKV sample solution was pipetted directly on the loading area of a paper microfluidic chip, which spontaneously flowed through the paper fibers via capillary action (Fig. 1b). Once the solution reached the detection area, presumably with RNA targets but not with large proteins and cell fragments, it was excised (punch-holed) for amplification, RT-LAMP reaction mixture was then added to the detached detection area. The paper platform was then sandwiched and sealed between glass slides again to prevent evaporation, and placed onto a 68 °C hot plate (Fig. 1c). For each RT-LAMP assay on the hot plate, color change was monitored *in situ* by capturing a series of digital images using a smartphone camera, at 5, 10, 15, 30, and 40 minutes. The progression of amplification changes the color on the paper from yellowish-red (red with weak green) to yellow (red + green) (Fig. 3a). The acquired images were split into three color channels (RGB), and the individual color intensities were obtained from the detection area of each paper microfluidic chip. The raw RGB intensities, shown in Supplementary Table S1, depicted large increases in green intensities, followed by small decreases in blue intensities and consistently high red intensities with respect to the overall brightness. Therefore, the green intensity was used as a measure of the progression of amplification, while the red intensity was used as a reference, i.e. green intensity normalized with red intensity (G/R) throughout this study. As shown in Fig. 3b, G/R (average from three different experiments) remained constant up to 5 min of reaction, followed by a dramatic increase between 5 to 15 min, and finally a saturation after 20 min. Gel electrophoresis was also performed for these samples, again by eluting the amplicons using TE buffer and subsequent sonication to confirm real amplifications, as shown in Fig. 3d. While successful amplification was confirmed only after 30 minutes with gel electrophoresis, it can be confirmed as early as 10 minutes on a paper microfluidic chip (*p* < 0.05), demonstrating the superiority of this method. For the remaining paper microfluidic RT-LAMP assays, 15-minute assay time was used to ensure sufficient amplification, while 10-minute time was used for the specificity assays where excess amplifications were unnecessary and should be avoided.

### Specificity of assay

**Figure 4:**
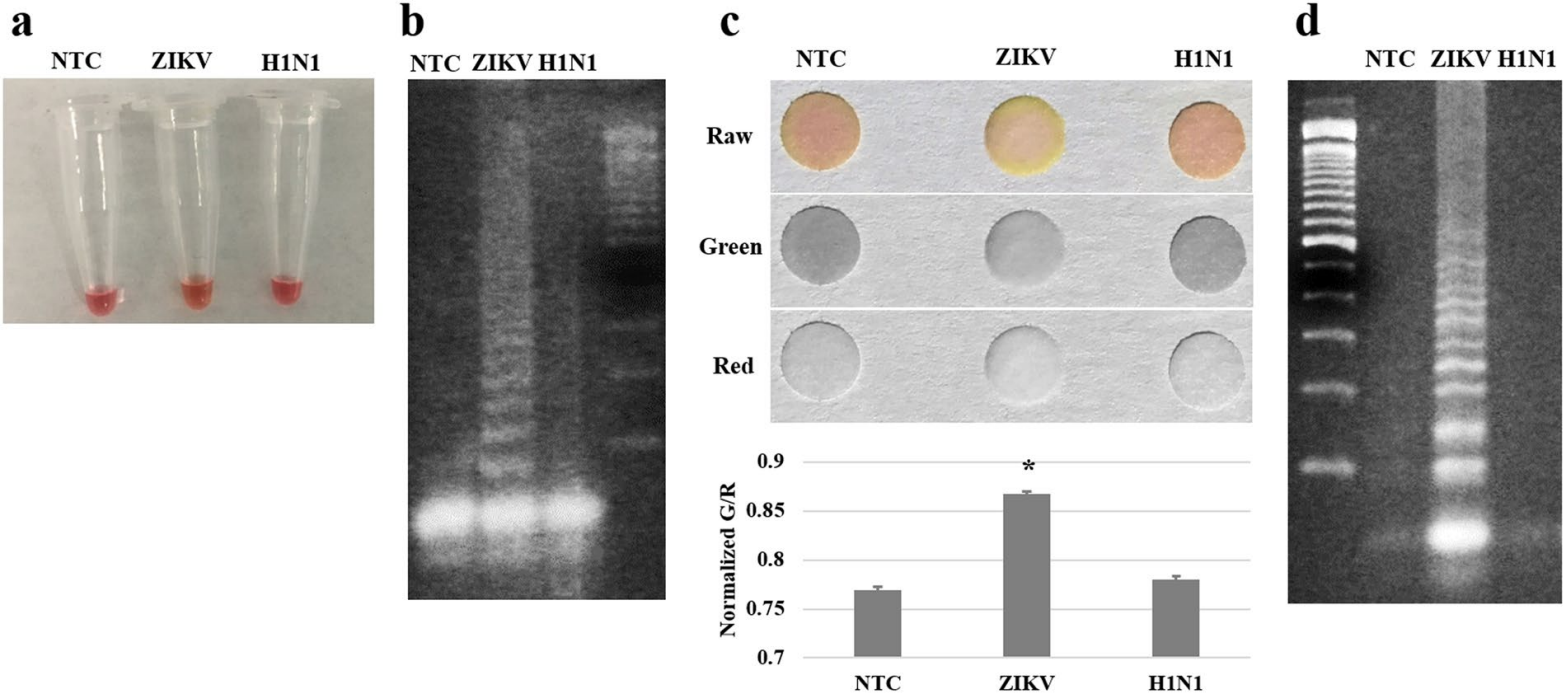
Specificity assays with conventional RT-LAMP **(a,b)** and paper microfluidic RT-LAMP **(c,d)**. Both ZIKV and influenza A/H1N1 samples were assayed using ZIKV primers. **(a,b)** Conventional RT-LAMP as confirmed with the 3% w/v agarose gel electrophoresis, showing almost no difference in color development. **(c,d)** Paper microfluidic RT-LAMP (representative smartphone images) as confirmed with the 3% w/v agarose gel electrophoresis, showing substantial difference in color development with ZIKV over NTC and H1N1 (bar charts show the average G/R intensity values from thee different experiments, each using a different paper microfluidic chip, sample, and reagent. Error bars represent standard errors).

Influenza A/H1N1 (swine flu) was used to examine the specificity of this assay using the same three sets of primers for ZIKV. These primers were designed to specifically bind to the NS5 gene sequence in ZIKV^20^. Both H1N1 and ZIKV are single-stranded RNA viruses, with similar genome size: 13,498 bases long for H1N1 virus^21^ and 10,676 bases long for Zika virus^22^. RT-LAMP assays were conducted using a colorimetric dye on the paper microfluidic chips sitting on a heated hot plate. Assay time was set to 10 min, following the results of real-time monitoring and assay time (Fig. 3). A conventional thermocycler was also used for comparison purposes. The results shown in Fig. 4 confirm that the paper microfluidic RT-LAMP assay shows high specificity, similar to conventional RT-LAMP in a thermocycler, i.e. strong amplification of ZIKV and no amplification from NTC and H1N1 as confirmed by the G/R values (averages from three different experiments) and gel electrophoresis.

### Limit of detection

**Figure 5:**
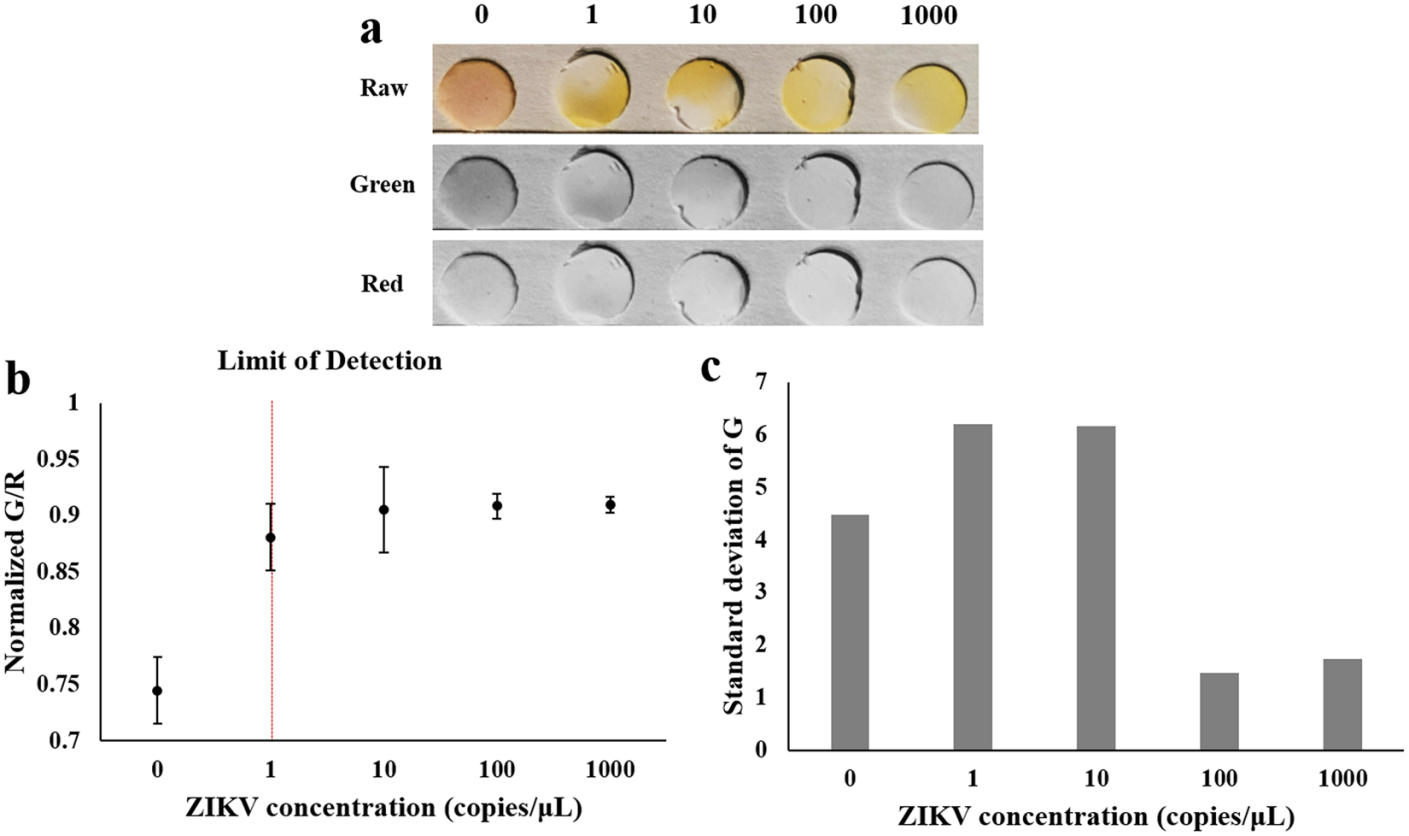
Limit of detection (LOD) with paper microfluidic RT-LAMP. **(a)** Representative, raw, green and red channel smartphone images of paper chips with varied ZIKV concentrations from 1 to 1000 copies/µL. **(b)** Plot of normalized green to red (G/R) intensity against ZIKV concentration, showing the LOD of 1 ZIKV copy/µL using the paper microfluidic RT-LAMP. Average of three different experiments, each using a different paper microfluidic chip, sample, and reagent. Error bars represent standard errors. **(c)** Standard deviations of green intensities across the detection areas of the paper microfluidic chips, plotted against ZIKV concentration.

ZIKV samples were serially diluted from 10^3^ copies/µL down to 1 copy/µL (i.e., the lowest possible copy number). The preceding paper microfluidic RT-LAMP procedures were then repeated utilizing these various concentrations. At 15 minutes, yellow coloration developed on paper for all ZIKV concentrations, including 1 copy/µL, as shown in Fig. 5a. Circular crops were used to evaluate the green and red intensities from the detection areas of the paper chips. These normalized G/R values were plotted against the initial virus concentration, as shown in Fig. 5b. The normalized G/R values for all four ZIKV concentrations were statistically different from that of a control (*p* < 0.05), indicating that 1 copy/µL is the limit of detection for this smartphone-based and paper microfluidic RT-LAMP assay for ZIKV. At lower ZIKV concentrations (1 and 10 copies/μL), however, such yellow colorations were not evenly distributed, as indicated by relatively high standard deviations of green intensities from the circular detection areas (Fig. 5c)^23^. At higher ZIKV concentrations (100 and 1000 copies/μL), yellow colorations became relatively evenly distributed with lower standard deviation values (Fig. 5c).

### Sample matrices

ZIKVs are generally found in the bodily fluids of infected patients such as urine and blood^24^. Additional purification steps are necessary to conduct nucleic acid amplification assays (including LAMP) of ZIKV from urine and blood samples. The use of a smartphone and paper microfluidic chips demonstrated in this work provides the possibility of field- and clinic-based, rapid and easy-to-use diagnostics. Smartphone and paper-based RT-LAMP assays were repeated using human urine (undiluted) and human blood plasma (diluted to 10% v/v with Plasma-Lyte) samples spiked with ZIKV. Tap water (undiluted), again spiked with ZIKV, was also tested in this work since it has been considered another possible route for ZIKV transmission^25^.

**Figure 6:**
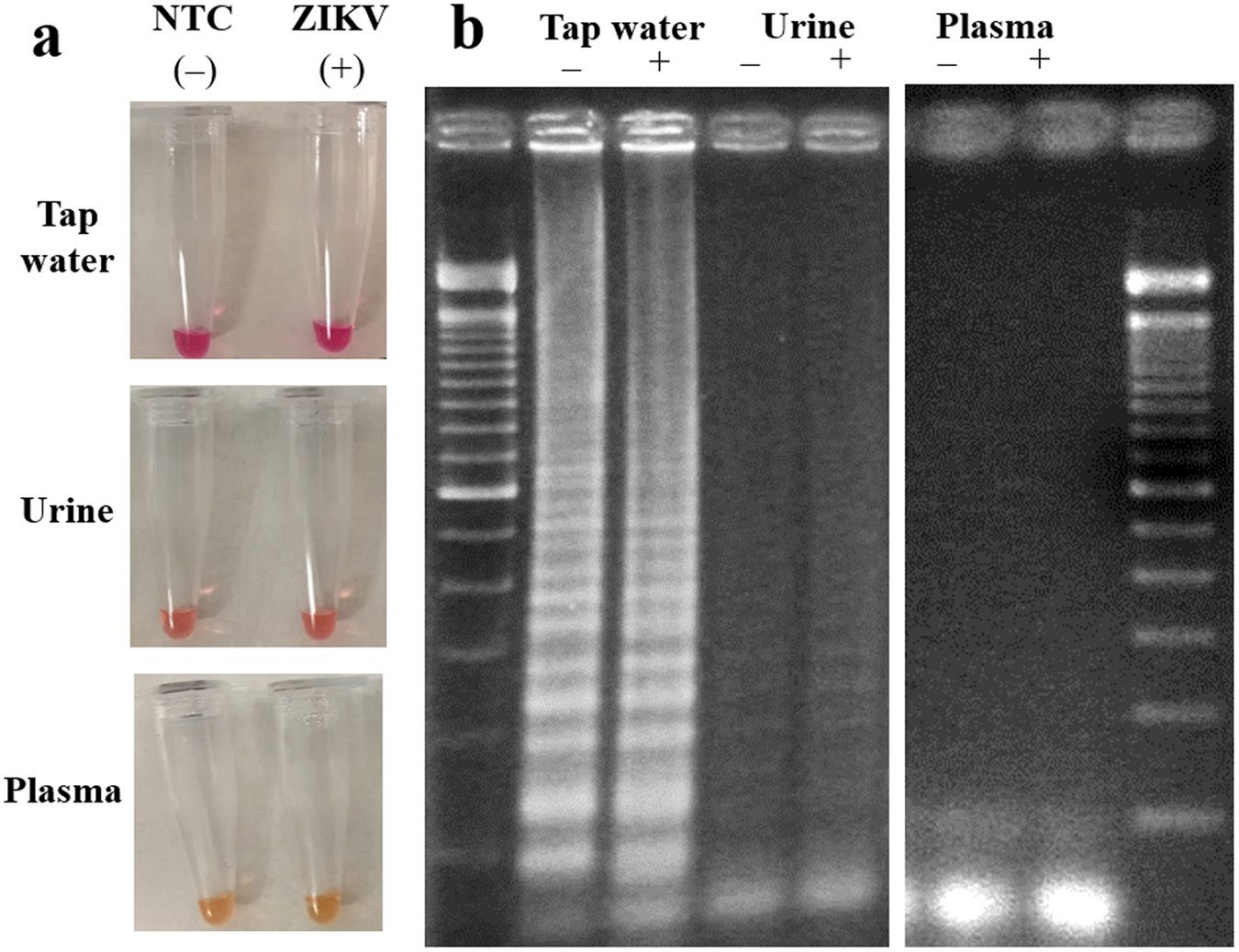
The effect of sample matrices to conventional RT-LAMP. ZIKV was spiked into tap water (undiluted), human urine (undiluted), and human blood plasma (diluted to 10% using PlasmaLyte), with final concentration of 10 copies/μL. **(a)** Images of reaction tubes after 15 minutes of reaction. Yellow coloration indicates successful amplification, i.e., all of them are not successful. **(b)** The gel images after 15 minutes of reaction, again showing either non-specific amplification (tap water) or no amplifications (urine and plasma).

First, conventional RT-LAMP assays were conducted for tap water, urine, and plasma samples spiked with ZIKV (final concentration = 10 copies/μL in the sample). The volume of spiked ZIKV solution was very small (0.5 μL out of 50 μL; thus 1% v/v) and thus samples were virtually undiluted. To these samples, primers and colorimetric RT-LAMP master mixes (15 μL) were added without any filtration process. Color changes from the tubes are shown in Fig. 6a and the gel electrophoresis results are shown in Fig. 6b. With the tap water sample, no clear color change (i.e. yellow coloration) was observed, even though tap water is considered relatively clean. Both target (+) and NTC (−) samples were amplified as shown in the gel electrophoresis, although both are non-specific amplifications considering the band locations (100, 150, 200, 250, 300, 350, 400, 450, 500, 600, 700 and 800, i.e., apparently a mixture of at least two different amplified products). Tap water might have contained contaminant that could be amplified non-specifically. However, the coloration in the tube did not change to yellow (indicating acidic condition, pH < 6.8) despite the presence of non-specifically amplified products (negatively charged), requiring alternative explanation. More likely, cations in high ionic strengths common in tap water (especially calcium and magnesium) might have falsely raised the pH from acidic to neutral despite the presence of amplified nucleic acids. This hypothesis could further be supported by the bright pink color in the tube, indicating strong alkaline condition (pH > 8). In addition, cations in very high ionic strength might have destabilized the contaminated, double-stranded DNA, and affect the stringency of primer annealing, leading to non-specific amplification^26^.

With urine and plasma samples, the color did not change upon amplification; it remained orange with urine and yellow with plasma, presumably due to the yellow coloration of urobilin in urine and the weak yellow coloration in plasma, respectively. However, neither specific nor non-specific amplifications were observed for both urine and plasma samples, as shown in the gel electrophoresis images. (Very weak signs of LAMP can be seen with urine sample, however.) A wide variety of protein molecules in urine and plasma inhibited the amplification processes such as polymerase activity and the binding affinity between primers and target strands^27^.

**Figure 7:**
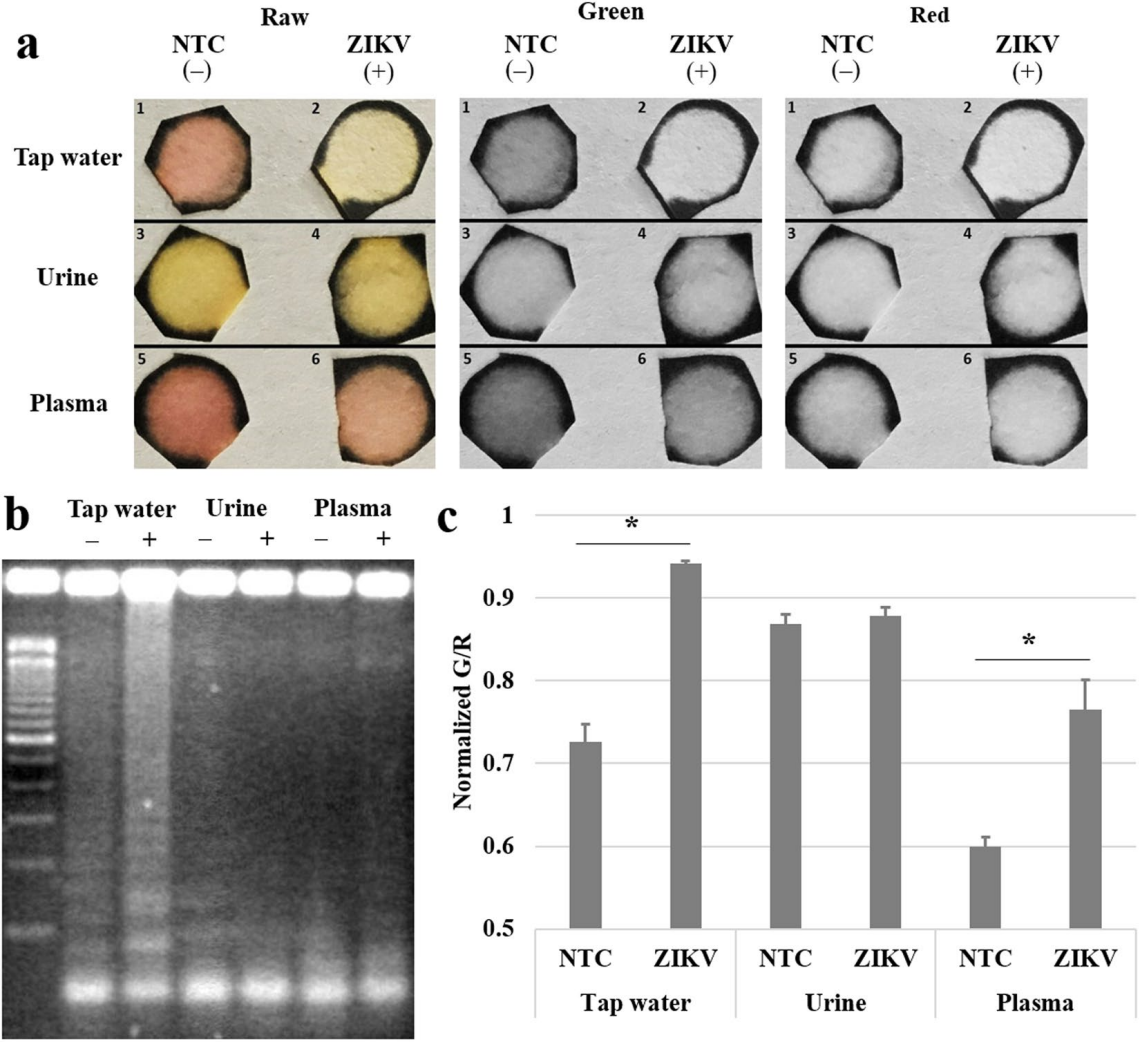
The effect of sample matrices to paper microfluidic RT-LAMP. ZIKV was again spiked into tap water (undiluted), human urine (undiluted), and human blood plasma (diluted to 10% using PlasmaLyte), with final concentration of 10 copies/μL. **(a)** Representative raw, green and red channel images of paper after 15 minutes of reaction, showing different colorations between positive and NTC samples. **(b)** The gel images after 15 minutes of reaction show non-successful amplifications, indicating the amplification time was not long enough or the elution from paper was not optimum. **(c)** Normalized green to red (G/R) intensity values were obtained from smartphone images, indicating successful distinction between positive and NTC samples, thus superiority of this smartphone-based assay. Average of three different experiments, each using a different paper microfluidic chip, sample, and reagent. Error bars represent standard errors.

On the other hand, our paper microfluidic RT-LAMP assay showed significant improvement over the aforementioned conventional RT-LAMP. The green and red pixel intensities, extracted from the smartphone images, showed statistical differences between ZIKV-spiked samples (10 copies/μL; +) and NTC (−) with tap water and plasma (*p* < 0.05) (Fig. 7). Tap water samples showed the greatest differences between target (+) and NTC (–). Cellulose fibers in paper should be able to filter large-sized particulate matters in the samples^28,29^ and the overall negative polarity of cellulose could compensate for the adverse effects caused by cations. As described previously, cations in high concentrations common in tap water and plasma can falsely raise the pH, negatively affecting the color development of a pH indicator dye as well as the stringency of primer annealing and subsequent LAMP reactions. With paper chips, the negative polarity as well as the weak negative charge of cellulose fibers (which are weakly carboxylated) might be able to neutralize this false pH raise, at least in part, leading to correct yellow coloration and subsequently successful amplifications. These negative polarity of paper fibers also repels negatively charged target RNA, allowing target RNA to flow through the pores at the same speed as that of bulk capillary action. It also prevents non-specific adsorption of primers and amplified products to paper fibers during amplification. Most importantly, larger protein molecules and cell fragments in urine and plasma samples travel at a much slower speed (retention), leading to successful filtration and amplification on paper microfluidic chips. It is also possible that all three samples (tap water, urine, and plasma) might have contained non-specifically amplified LAMP products of high molecular weights via cross-contaminations, which may be responsible for the non-specific amplification with tap water using conventional LAMP. These contaminated LAMP products could have been successfully filtered during the paper microfluidic filtration step.

Such distinction can also be found with urine sample, although without statistical significance (*p* > 0.05), which can be attributed to the strong, yellow coloration of urobilin in urine. While plasma is also yellow in color, its yellow coloration is much weaker than that of urine, leading to successful distinction, although it is still inferior to that of tap water.

In addition, sample filtration, subsequent amplification, and real-time quantification were all performed on a single paper microfluidic chip, without any liquid transfers, which reduced the possibility of introducing the environmental contaminants. In the conventional LAMP assay shown in Fig. 6, the non-specific amplification might have originated from the laboratory environment during the multiple liquid transfer steps.

These results were compared with agarose gel electrophoresis, using the samples eluted from paper chips. The results were generally improved over conventional RT-LAMP, including substantial difference between target (+) and NTC (−) with tap water, some product formation with plasma, et cetera. However, since the amplification time (15 minutes) is too short for gel electrophoresis, the results are not as convincing as the colorimetric assays, indicating the superiority of the paper microfluidic RT-LAMP.

## Conclusions

A simple, rapid and real-time paper microfluidic assay coupled with smartphone detection was demonstrated for detecting ZIKV from complex sample matrices through RT-LAMP. The developed method requires paper microfluidic chips (easily printed using a wax printer), a hot plate, and a smartphone, along with the necessary RT-LAMP reaction mixture – rendering this method as potentially useful in clinical and field settings, especially in resource-limited areas. Amplification could be quantified in real-time and statistically significant pixel intensities could be obtained as early as 10 minutes. The limit of detection was 1 genome copy per μL. Amplification was also successful in the presence of sample matrices (undiluted tap water, undiluted urine, and 10% diluted human blood plasma), due to the filtration capability of cellulose fibers during capillary action, as well as conducting sample filtration, amplification, and real-time quantification on a single paper microfluidic chip without any liquid transfers. This method can be easily adapted for detecting other types of viral or bacterial targets. Precautions should be taken during the field-based assays to prevent cross-contamination between tests, i.e., changing pipette tips, cleaning punch holes, and cleaning the hot plate surface after each assay. If further analyses such as gel electrophoresis and/or sequencing are necessary, any stray amplicons must be digested prior to each assay in an exceptionally sanitized laboratory. Future work includes the development of a software application on a smartphone allowing automated and user-friendly data analysis, together with the use of a negative control channel to compensate for ambient lighting bias.

## Methods

### Primers and RT-LAMP master mix

LAMP primers were chosen from the literature^20^. The primer mixtures were diluted to 2 μM each of F3 (forward outer primer), 5′-CGGATGGGATAGGCTCAAAC-3′, and B3 (backward outer primer), 5′-ATGGACCTCCCGTCCTTG-3′, 16 μM each of FIP (forward inner primer), 5′- CCTGAGGGCATGTGCAAACCTAGAATGGCAGTCAGTGGAGAT-3′, and BIP (backward inner primer), 5′-ACCCTCAACTGGATGGGACAACTGGAGCTTGTTGAAGTGGTG-3′, and 4 μM each of LoopF (forward loop primer), 5′-CATCAATTGGCTTCACAACGC-3′, and LoopB (backward loop primer), 5′- GGGAAGAAGTTCCGTTTTGCTC-3′. The 2X DNA & RNA WarmStart colorimetric LAMP master mix (New England Biolabs, Ipswich, MA, USA) consisted of *Bst* 2.0 WarmStart DNA Polymerase, WarmStart RTx, 16.0 mM MgSO_4_, and phenol red as a pH indicator dye. The RT-LAMP final mixture contained 6.25: 1.25: 2.5 ratio of 2X colorimetric RT-LAMP master mix: primer mixture: target (or nuclease-free water for no target control, NTC).

### Sample preparation

Zika virus (ZIKV) NATtrol (high concentration range verification panel; Zeptometrix, Buffalo, NY, USA) was extracted using the Quick-DNA/RNA Miniprep kit and its protocol (Zymo Research, Irvine, CA, USA). The initial copy number was 10^3^ copies/μL, which was also serially diluted to 10^2^, 10, and 1 copies/μL. Influenza A/H1N1 NATtrol (external run control medium; Zeptometrix, Buffalo, NY, USA) strain A/NY/02/2009 was extracted using the Quick-DNA/RNA Miniprep kit and its protocol. The initial copy number was 10^2^ copies/μL. The extracted ZIKV RNA was spiked into the tap water (undiluted), human urine (undiluted) and human blood plasma (diluted to 10% using PlasmaLyte, Baxter International Inc., Deerfield, IL, USA) samples at 1% v/v ratio so that the samples were virtually undiluted. Tap water was obtained from the Biosensors Laboratory at the University of Arizona. Human urine was purchased from Lee Biosolutions (St. Louis, MO, USA), which is normal urine from pooled human donors and is pathogen-free. Human blood plasma was purchased from ZenBio, Inc. (Research Triangle Park, NC, USA), which is normal plasma and pathogen-free. All experiments were performed in the Biosensors Laboratory at the University of Arizona, which is at biosafety level 2 and chemical safety level 2 and in accordance with relevant guidelines and regulations. Since the sample collection was performed by the vendors and the urine and plasma samples were pathogen-free, approval by the institutional review board was waived.

### Temperature optimization

The optimum temperature was evaluated by conventionally amplifying the NTC and target at three different temperatures, 65 °C, 68 °C, and 70 °C for 30 minutes using a thermocycler (MJ Research, Waltham, MA, USA), followed by refrigeration at 4 °C in preparation for gel electrophoresis confirmation.

### Paper type optimization

Multiple paper types were tested for optimizing the paper microfluidic RT-LAMP assay. Cellulose chromatography papers (Whatman, Pittsburgh, PA, USA) of grade 1 (pore size of 10 µm) and grade 4 (pore size of 20–25 µm) were evaluated, as well as nitrocellulose (NC) paper (pore size of 0.3 µm) (Millipore, Burlington, MA, USA).

### Design and fabrication of paper microfluidic chips

The microfluidic chip was designed using SolidWorks 2015 software (SolidWorks Corp., Concord, MA, USA). The chip design consisted of the 5 × 5 mm sample loading area, connected to a 3 × 30 mm main channel (where filtration occurred), and a 5 mm diameter circular end (detection area) where target RNA was collected (Fig. 1a). Channel design was printed on an optimized type of chromatography paper using a wax printer (Colorqube, Xerox, Norwalk, CT, USA). The printed wax was reheated using a hot plate set at 120 °C for 5 minutes to create hydrophobic barriers throughout the depth of paper to complete fabrication.

### Sample loading and filtration on paper microfluidic chips

50 µL of ZIKV-spiked samples in various matrices (water, undiluted tap water, undiluted human urine, and 10% diluted human blood plasma) were pipetted onto the sample loading area of each paper microfluidic chip. The solution spontaneously flowed along the main channel via capillary action, and bulky biomolecules were filtered out as early as at the beginning of the channel, while ZIKV RNAs passed through with the liquid towards the circular end (detection area) of a chip. The dimensions of loading and detection areas were optimized to sufficiently accommodate the volume of sample (50 μL). As for the length of main channel, longer channels increased the filtration effect. However, due to the evaporation during capillary action, there exists a certain optimum length for the given volume of sample, which was determined to be 30 mm through a series of trial-and-error experiments. The circular end (detection area) of the chip was excised and placed on a hot plate in preparation for the amplification and subsequent detection, which is described in the following section.

### RT-LAMP on paper microfluidic chip

The detection area of each paper microfluidic chip was excised by hole-punching it into 5-mm diameter circles and placed on a glass slide (Amscope, Irvine, CA, USA). The hole-puncher was cleaned with RNase AWAY decontamination reagent (Life Technologies, Carlsbad, CA, USA) after each assay, in order to prevent cross-contaminations. 15 µL RT-LAMP reaction mixture was loaded on this excised paper, and covered with another glass slide to sandwich the paper, followed by sealing with Parafilm M (Bemis, Neenah, WI, USA) to prevent evaporation. RT-LAMP mixture consisted of 12.5 µL colorimetric master mix and 2.5 µL primers. The paper sandwiched between glass slides was placed on a hot plate with a feedback temperature control at 1 °C resolution (MS-H-Pro, SCILOGEX, Rocky Hill, CT, USA), set at 68 °C for 30 minutes.

### Confirmation with gel electrophoresis

For the purpose of gel electrophoresis confirmation, the amplicons were eluted out of the paper by submerging it in 50 µL Tris-HCl and EDTA (TE) buffer (Ambion, Foster City, CA, USA), followed by sonication for 30 minutes in a sonicate bath (Branson, Danbury, CT, USA). Amplification was confirmed with gel electrophoresis using 3% w/v agarose gel (Sigma-Aldrich, St. Louis, MO, USA) and 1X Tris-acetate-EDTA (TAE) buffer (Invitrogen, Carlsbad, CA, USA).

### Real-time quantification using a smartphone

During amplification, the images of paper were captured using a smartphone camera (CMOS array) under ambient lighting conditions. Images were captured at 5, 10, 15, 30, and 40 minutes after the onset of amplification. Raw images were split into RGB images and the red, green and blue intensities were evaluated over the area of interest (the entire circular detection area of each paper microfluidic chip) using ImageJ software (U.S. National Institutes of Health, Bethesda, MD, USA). The red and green intensities were averaged over the detection area and the standard deviations were quantified from three different experiments. For the limit of detection experiments shown in Fig. 5, the distributions of green pixel intensities were additionally evaluated by randomly selecting 10 different oval shapes of equal size (2.66 mm by 2.57 mm) from the detection area of each paper microfluidic chip. With three different experiments, a total of 30 small oval shapes were obtained and their averages and standard deviations were evaluated to address the distribution of green colorations.

## Electronic supplementary material

Supplementary Information

## Data Availability

All data generated or analyzed during this study are included in this published article.
