# Supplementary material for: Simpler, Faster, and Sensitive Zika Virus Assay Using Smartphone Detection of Loop-mediated Isothermal Amplification on Paper Microfluidic Chips
Source: Sci Rep. 2018 Aug 20;8:12438. doi: 10.1038/s41598-018-30797-9 (PMC6102244; doi:10.1038/s41598-018-30797-9)
Supplement: Supplementary file 1 — Supplementary Information [file 41598_2018_30797_MOESM1_ESM.docx]

**Simpler, Faster, and Sensitive Zika Virus Assay Using Smartphone Detection of Loop-mediated Isothermal Amplification on Paper Microfluidic Chips**

Kattika Kaarj^1^, Patarajarin Akarapipad^2^, and Jeong-Yeol Yoon^1,2^

**Supplementary Figure S1.** The raw agarose gel electrophoresis images used in Fig. 1. a) Paper type optimization among NC (nitrocellulose), G4 (cellulose grade 4) and G1 (cellulose grade 1) papers. Lane 1 ˗ 8 are not related to this work. They are the results of similar project which is done in our group. b) Temperature optimization among 65°C, 68°C and 70°C using a conventional thermocycler and the replication of paper type optimization among NC (nitrocellulose), G4 (cellulose grade 4) which cannot be clearly seen in a). Lane 1 and 2 are not related to this work.

**
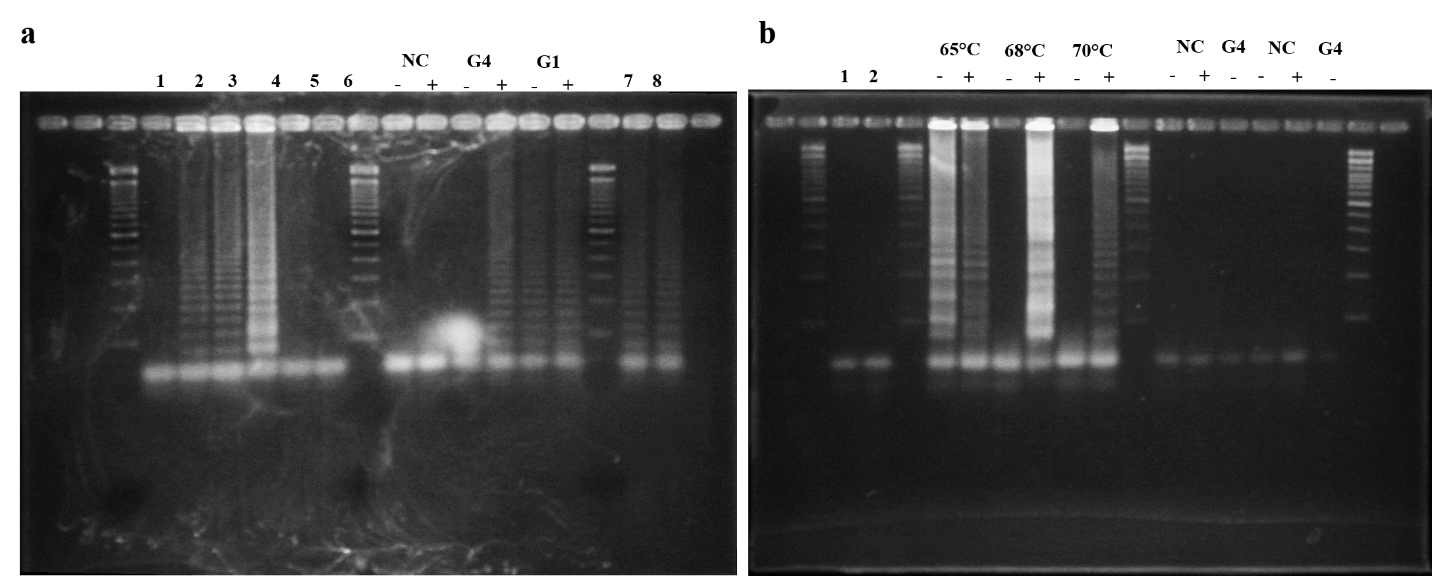
**

**Supplementary Figure S2.** The raw agarose gel electrophoresis images used in Fig. 3d.

**
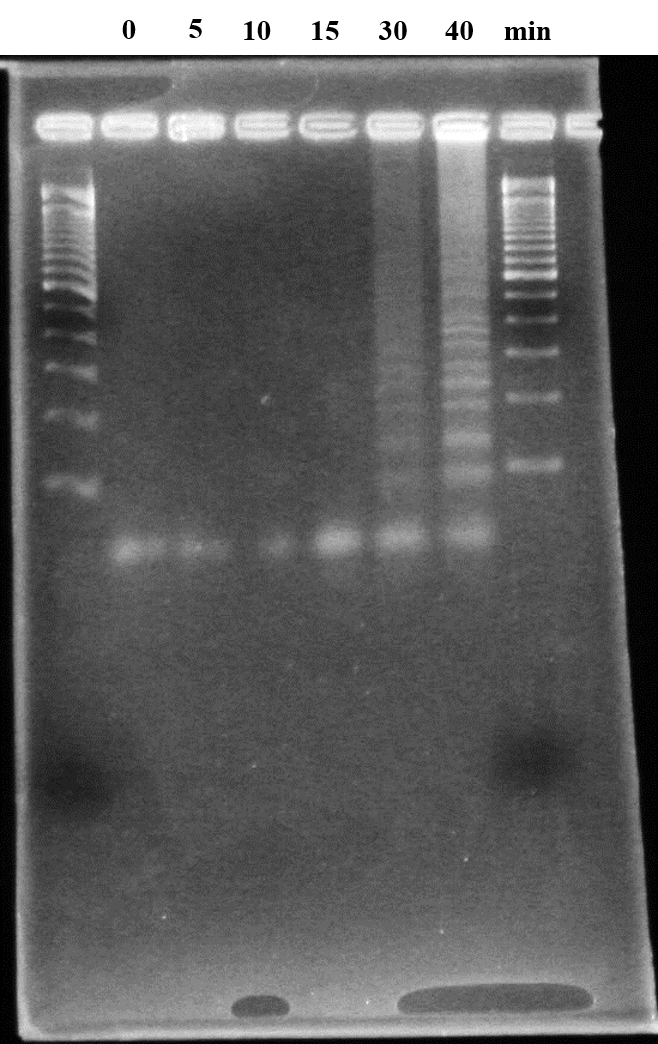
**

**Supplementary Figure S3.** The raw agarose gel electrophoresis images used in Fig. 4b and 4d.


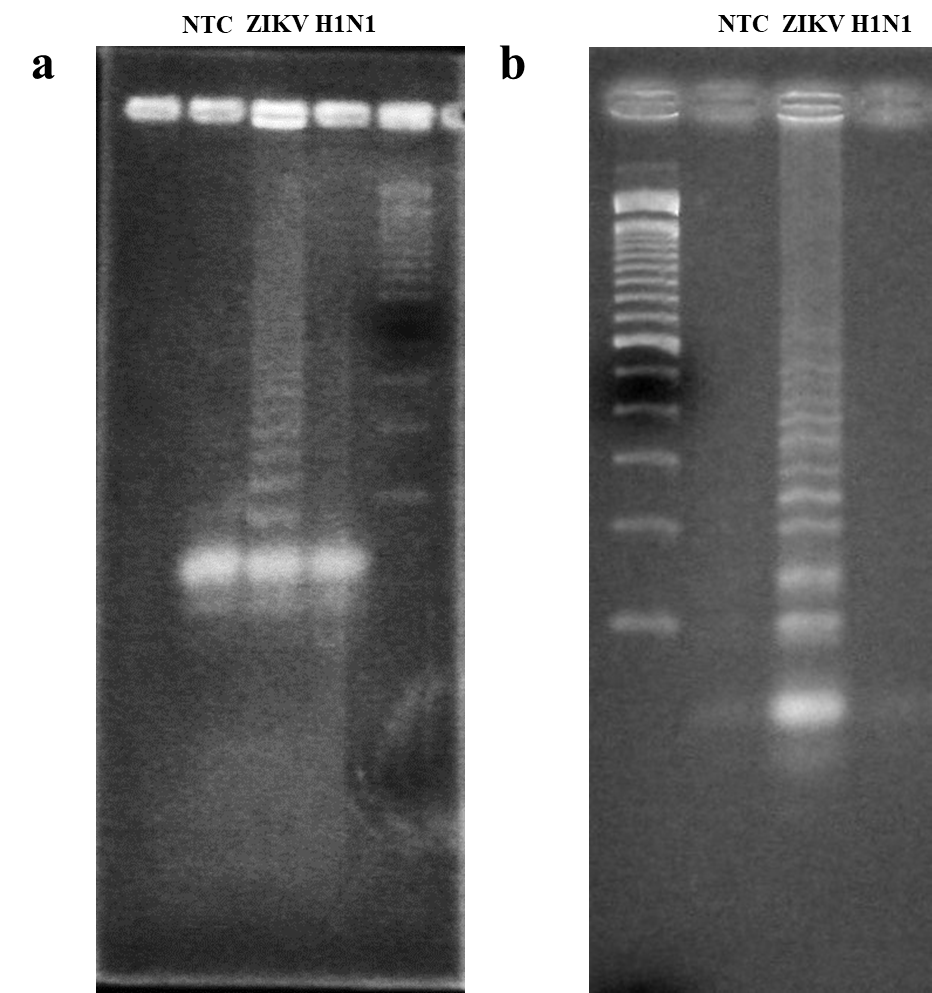


**Supplementary Figure S4.** The raw agarose gel electrophoresis images used in Fig. 6b. a) The ZIKV was spiked into undiluted tap water, urine and diluted human blood plasma. The non-specific amplification presented within human blood plasma sample because of the non-specific temperature within the different location in thermocycler. b) The replication of the human whole blood sample with the controlled specific temperature.

**
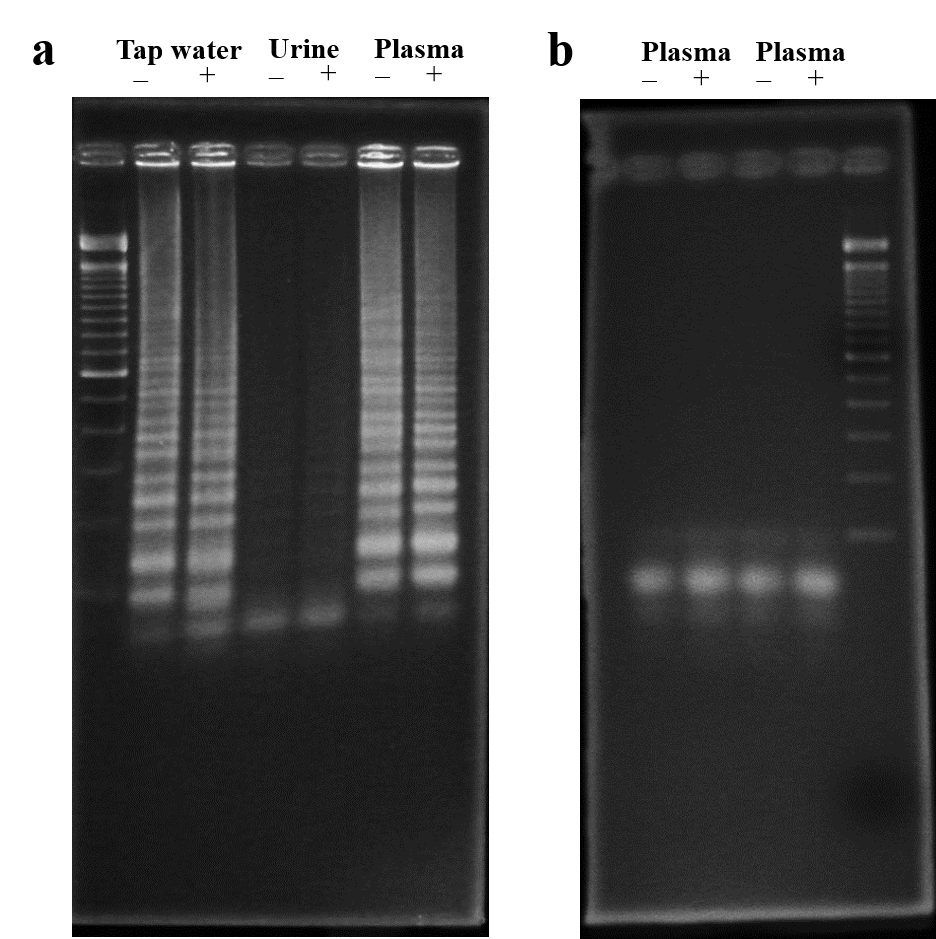
**

**Supplementary Figure S5.** The raw agarose gel electrophoresis images used in Fig. 7b.

**
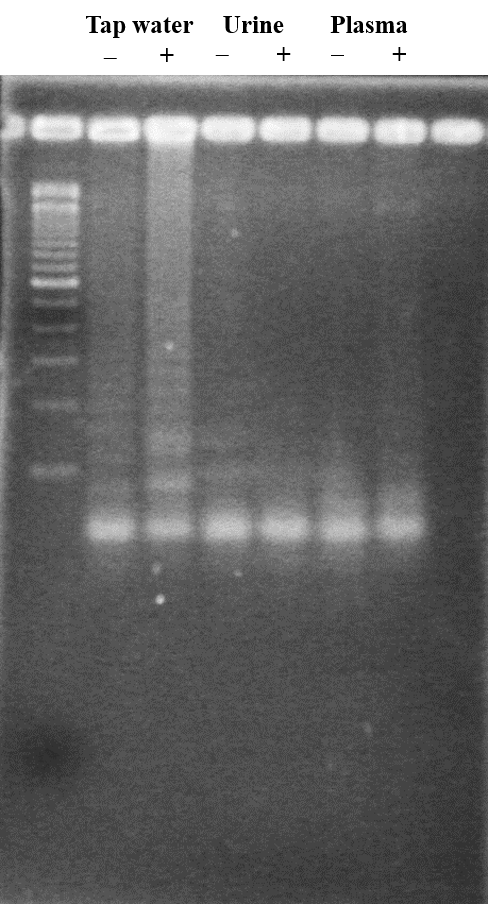
**

**Supplementary Figure S6.** The channel length was optimized from 5, 10, 20 and 30 mm by evaluating the colorations from paper microfluidic LAMP. Yellow coloration, i.e. successful amplification, could be observed only with the 30-mm channel, indicating ZIKV RNAs successfully reached the detection area and amplification inhibitors (contaminants) were efficiently filtrated. Red colorations with shorter channels (5, 10 and 20 mm) indicate unsuccessful amplification and potentially inefficient filtration.


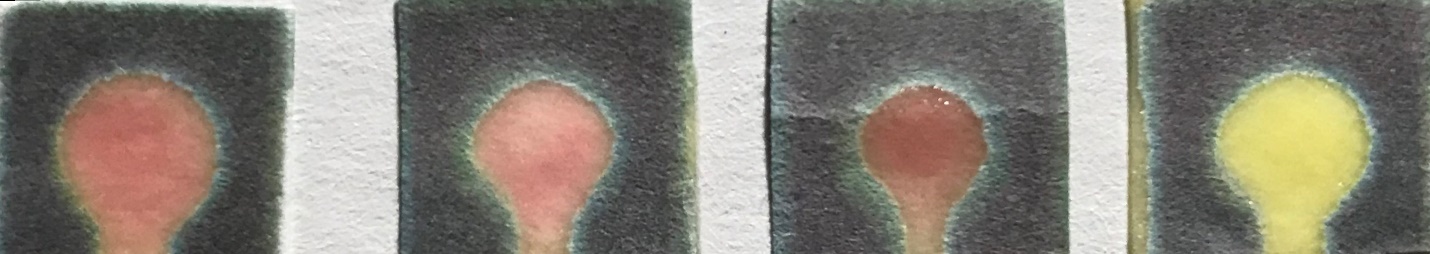


**30 mm**

**20 mm**

**10 mm**

**5 mm**

**Supplementary Table S1.** Red, green, and blue intensities from the detection areas of paper chip. Experiments were repeated three times, each time using a different paper chip. G/R = (average G) / (average R).

For Figure 3 (real-time monitoring)

| Assay time | Intensities | | | | | | | | | |
| --- | --- | --- | --- | --- | --- | --- | --- | --- | --- | --- |
|  |  | R |  |  | G |  |  | B |  | G/R |
| 0 min | 153 | 156 | 156 | 117 | 113 | 114 | 87 | 88 | 84 | 0.740 |
| 5 min | 168 | 184 | 173 | 134 | 123 | 130 | 98 | 85 | 92 | 0.739 |
| 10 min | 180 | 173 | 175 | 151 | 144 | 146 | 106 | 97 | 92 | 0.835 |
| 15 min | 197 | 192 | 193 | 170 | 167 | 171 | 114 | 103 | 110 | 0.873 |
| 30 min | 212 | 215 | 211 | 194 | 199 | 191 | 135 | 129 | 137 | 0.915 |
| 40 min | 215 | 214 | 216 | 203 | 197 | 199 | 131 | 137 | 139 | 0.929 |

For Figure 4 (specificity)

| Sample | Intensities | | | | | | | | | |
| --- | --- | --- | --- | --- | --- | --- | --- | --- | --- | --- |
|  |  | R |  |  | G |  |  | B |  | G/R |
| NTC | 194 | 194 | 198 | 150 | 149 | 152 | 126 | 128 | 129 | 0.770 |
| ZIKV | 204 | 206 | 214 | 177 | 178 | 186 | 139 | 140 | 147 | 0.867 |
| H1N1 | 203 | 206 | 208 | 158 | 160 | 163 | 134 | 136 | 138 | 0.780 |

For Figure 5 (LOD)

| Copies  /µL | Intensities | | | | | | | | | |
| --- | --- | --- | --- | --- | --- | --- | --- | --- | --- | --- |
|  |  | R |  |  | G |  |  | B |  | G/R |
| 0  1  10  100  10000 | 198 | 193 | 196 | 141 | 145 | 151 | 99 | 107 | 103 | 0.745 |
|  | 197 | 200 | 198 | 174 | 170 | 180 | 117 | 108 | 131 | 0.881 |
|  | 204 | 207 | 209 | 189 | 192 | 180 | 120 | 111 | 144 | 0.905 |
|  | 211 | 213 | 210 | 192 | 191 | 193 | 137 | 118 | 130 | 0.909 |
|  | 207 | 205 | 207 | 187 | 188 | 188 | 108 | 126 | 108 | 0.910 |

For Figure 7 (sample matrices)

| Sample | Intensities | | | | | | | | | |
| --- | --- | --- | --- | --- | --- | --- | --- | --- | --- | --- |
|  |  | R |  |  | G |  |  | B |  | G/R |
| Tap –  Tap + | 195 | 201 | 191 | 145 | 141 | 140 | 115 | 111 | 112 | 0.726 |
|  | 216 | 215 | 217 | 203 | 202 | 205 | 162 | 157 | 162 | 0.941 |
| Urine –  Urine + | 199 | 200 | 200 | 175 | 174 | 171 | 87 | 89 | 89 | 0.868 |
|  | 195 | 197 | 198 | 169 | 175 | 174 | 96 | 99 | 100 | 0.878 |
| Plasma –  Plasma + | 178 | 182 | 179 | 107 | 111 | 105 | 81 | 81 | 82 | 0.599 |
|  | 198 | 197 | 191 | 146 | 148 | 154 | 112 | 118 | 110 | 0.765 |
